# Evaluating the Protective Effects of MitoQ and Antifreeze Protein III on Cryopreserved Canine Sperm

**DOI:** 10.3390/ani15020270

**Published:** 2025-01-19

**Authors:** Abbas Farshad, Emilia Diel, Axel Wehrend

**Affiliations:** Veterinary Clinic for Reproductive Medicine and Neonatology, Justus-Liebig-University of Giessen, 35392 Giessen, Germany; emilia.diel@vetmed.uni-giessen.de (E.D.); axel.wehrend@vetmed.uni-giessen.de (A.W.)

**Keywords:** mitoQ, antifreeze protein III, freezing, dog, spermatozoa

## Abstract

Cryopreservation can adversely affect sperm motility, structural integrity, and fertilization ability. This study investigated the effects of MitoQ and antifreeze protein III (AFP III) on frozen–thawed semen from eight adult dogs using a Tris–fructose extender. Ejaculates were divided and diluted with a standard Tris–fructose–egg yolk extender containing MitoQ (200 nM/mL) and AFP III (0.75, 1.0, 2.0 µg/mL), individually or combined. Post-thaw, samples were evaluated for motility, viability, membrane and acrosome integrity, lipid peroxidation, apoptosis indicators, mitochondrial function, and reactive oxygen species (ROS-H_2_O_2_). The results showed significant (*p* < 0.05) improvements in motility rate, progressive motility, VAP, VSL, VCL, ALH, and BCF with MitoQ or AFP alone. AFP III (0.75, 1.0 µg/mL) showed higher values than controls (*p* > 0.05), while MitoQ alone showed no significant effect. Viability and acrosome integrity improved with AFP III. Membrane integrity and lipid peroxidation were better in 0.75 and 1.0 µg/mL AFP III groups. ROS-H_2_O_2_ levels and mitochondrial membrane potential were unaffected except at 1.0 µg/mL AFP III. The phosphatidylserine translocation assay showed no significant differences in dead sperm between controls and individual treatments, but significant differences occurred with combined MitoQ/AFP III. In conclusion, AFP III and MitoQ in diluents protect canine sperm cells from cryodamage.

## Simple Summary

This study investigated the effects of MitoQ and antifreeze protein III (AFP III) on the frozen and thawed semen of dogs. The samples were mixed with solutions containing MitoQ (200 nM/mL) and AFP III (0.75, 1.0, and 2.0 µg/mL). After thawing, we observed significant improvements in sperm movement and structure with AFP III and MitoQ. AFP III also improved sperm viability and membrane integrity, especially at 0.75 and 1.0 µg/mL concentrations. There were no significant changes in ROS-H_2_O_2_ levels or mitochondrial function, except in the 1.0 µg/mL AFP III group. The combined treatment with MitoQ and AFP III significantly reduced dead sperm cells. In conclusion, AFP III and MitoQ can protect canine sperm from cryodamage.

## 1. Introduction

The freezing technique enables the long-term storage of sperm at extremely low temperatures; however, it also induces structural and functional damage. Research indicates that the low survival rate of sperm post-freezing is primarily attributed to ice crystal formation, osmotic pressure, lipid peroxidation, and plasma membrane alterations [1,2,3,4]. Despite its numerous advantages, sperm cryopreservation can significantly damage sperm cells, affecting their morphology, functionality, and overall viability [5]. This damage arises from complex processes, including ice formation, mechanical stress, oxidative stress, freeze dehydration, and ice recrystallization [6]. Cryoprotectants are critical in mitigating these effects, as they are incorporated into freezing solutions to minimize damage during cryopreservation [7,8]. Accordingly, the inclusion of cryoprotectants in extenders is vital for achieving successful sperm cryopreservation. In the last decade, substantial research has focused on antioxidant agents to mitigate or prevent cryodamage. Promising results have been achieved with various antioxidants, such as minocycline in rams [9]; trehalose and pentoxifylline in goats [10]; enzymatic- and non-enzymatic antioxidant in bulls [11]; sericin in mice [12]; silver nanoparticles in avians [13]; and coenzyme Q10 in stallions [14] during freeze–thaw processes.

In this context, coenzyme Q10, commonly referred to as Mitoquinone (MitoQ), is a well-researched antioxidant that specifically targets mitochondria. It comprises tetraphenylphosphonium (TPP) and ubiquinone [15]. These components are localized within the inner mitochondrial membrane and integrate into this structure, a process driven by the mitochondrial membrane potential [16,17]. MitoQ is found in high concentrations in the testis [18], where it plays a vital role in the electron transport chain involved in ATP synthesis and functions as a fat-soluble chain-breaking antioxidant [19,20,21]. Additionally, MitoQ protects against free radicals [18] and enhances sperm functionality by increasing energy production, which is essential for human sperm motility [22]. Due to its positive charge, MitoQ efficiently penetrates mitochondrial membranes and accumulates within the mitochondria [20,21]. Research has shown that MitoQ improves post-thaw viability, reduces lipid peroxidation in yellow catfish spermatozoa, and prevents testicular degeneration in mouse models. Furthermore, it promotes blastocyst development in bovine oocytes exposed to oxidative stress [23,24,25]. Mito-TEMPO, a mitochondria-targeted antioxidant that incorporates TPP (Triphenylphosphonium), has been documented to improve sperm quality and increase enzymatic antioxidant activity in thawed human sperm [26]. Similarly, Mito-TEMPO has also been reported to improve the quality of thawed semen in goats and roosters [27,28] and to increase the fertility of chilled and frozen–thawed ram semen [27,29,30].

Conversely, antifreeze proteins (AFPs), a specific group of polypeptides, are produced by certain insect species, bacteria, fungi, and polar fish [31]. These naturally occurring cryoprotectants perform multiple functions, including inducing thermal hysteresis, stabilizing cell membranes, and inhibiting ice crystal formation through a non-colligative mechanism [32,33,34,35,36]. Accordingly, the role of AFPs has been investigated using cryopreservation extenders initially developed to enhance the evaluation of sperm cells by examining various types and concentrations in different animal species [37,38,39,40]. The application of AFPs has also shown benefits in counteracting the harmful physical effects of ice recrystallization in red blood cells and preserving oocytes and embryos [41,42]. Moreover, AFPs help to maintain stable osmotic pressure around spermatozoa, which is crucial for keeping them in an inactive state. Consequently, the incorporation of AFPs reduces the need for high concentrations of penetrating chemical cryoprotectants, thereby lowering the toxicity associated with these agents [43,44].

To our knowledge, no studies have reported the use of MitoQ and AFP III for freezing canine sperm cells. Therefore, the main objective of this study was to evaluate the effects of these antioxidants on cryopreserved canine sperm. For this purpose, MitoQ and AFP III were used individually and in combination in diluents for freezing the canine sperm.

## 2. Materials and Methods

### 2.1. Reagents and Ethical Approval

The study utilized various chemicals and reagents obtained from the following companies: Sigma (Merck) in Darmstadt, Germany; Biozol Diagnostica Vertrieb GmbH in Eching, Germany; BioLegend in San Diego, CA, USA; and Hoelzel-Biotech in Koeln, Germany.

Ethical approval for the use of semen samples was granted by the local ethics authority through the animal welfare office at Justus Liebig University Giessen, as indicated by internal correspondence (IRB number kTV 11-2018).

### 2.2. Experimental Design and Procedures

This study was conducted at the Reproductive Medicine and Neonatology Departments of Justus Liebig University Giessen. Ejaculates were obtained using manual stimulation from healthy dogs with an average age of approximately 4 years, representing various breeds. Following collection, the semen samples were transported to the laboratory and placed in a water bath maintained at 37 °C for immediate analysis. Only samples from 8 dogs (*n* = 8), including Great Dane (*n* = 2), Border Collie (*n* = 1), Rough Collie (*n* = 1), German Shepherd (*n* = 2), and Labrador Retriever (*n* = 2), which demonstrated progressive motility above 70–75% and normal morphology above 70–75%, were selected for the study. The selected samples were divided into experimental groups and diluted with a basic extender containing 3.786 g Tris (hydroxymethyl-aminoethane), 2.172 g citric acid, 1 g fructose, 5.0% (*v/v*) glycerol, and 5.0% (*v/v*) egg yolk in 100 mL double-distilled water, to achieve a final concentration of 50–60 × 10^6^ sperm/mL. Treatment groups were supplemented with 200 nM mitoquinone mesylate (BYT-ORB1105367, BioLegend in San Diego, CA, USA), based on our previously established optimal concentrations and either 0.075 µg/mL or 1.0 µg/mL preformed antifreeze protein III (AFP III, CSB-EP360877FGV-Hoelzel-biotech, Hoelzel-Biotech in Koeln, Germany), both individually and in combination, based on previously established optimal concentrations. The control group received no additives.

The diluted samples were processed as follows: the samples were loaded into 0.25 mL straws and sealed at one end, then stored at 4–5 °C for 1.5 h. The samples were then frozen in nitrogen vapor, and positioned 4–5 cm above liquid nitrogen for 10 min. Finally, the samples were submerged in liquid nitrogen for storage lasting over 4 weeks, and each straw was thawed individually at 37 °C for 60 s prior to analysis.

### 2.3. Quality Assessment of Frozen–Thawed Semen

The motion characteristics of sperm were evaluated using the computer-assisted sperm motility analysis system (CASA: AndroVision^TM^ 3.5^®^, Minitube GmbH & Co, Tiefenbach, Germany), following the manufacturer’s guidelines for canine samples. Images were captured at a speed of twenty-five frames per second. To evaluate sperm motility and velocity, a 5 μL semen sample was placed on a pre-warmed chamber slide, covered with glass coverslips, and analyzed across 10 microscopic fields. This approach ensured that at least 2000 sperm were counted per sample. The parameters obtained from CASA included total motility (%), progressive motility (%), VAP (average path velocity, μm/s), VSL (straight linear velocity, μm/s), VCL (curvilinear velocity, μm/s), STR (straightness, VSL/VAP, (%), LIN (linearity, VSL/VCL, %), ALH (amplitude of lateral head displacement, μm), and BCF (beat cross frequency, Hz).

Sperm viability was determined using a modified eosin stain procedure [45], prepared by dissolving 2 g of eosin-B and 3 g of sodium citrate in 100 mL of distilled water. For this assay, a 5 μL aliquot of diluted sperm was placed on a pre-warmed slide, mixed with 10 mL of eosin solution, and allowed to air dry at room temperature. An optical microscope at 400× magnification was used to examine 200 sperm. Dead sperm were identified by pink or red heads, while live sperm had white heads. The results were expressed as a percentage.

Acrosome integrity was evaluated using a modified formalin–citrate solution [46]. In this procedure, 10 μL of semen was mixed with 100 μL of a formalin–citrate solution (composed of 96 mL of 2.9% sodium citrate and 4 mL of 37% formaldehyde). A portion of this mixture was transferred to a microscope slide and analyzed under a coverslip. A phase-contrast microscope at 1000× magnification was used to count 200 spermatozoa across at least three microscopic fields per sample. The percentage of spermatozoa with intact acrosomes was determined, and the results were expressed as a percentage.

The hypo-osmotic swelling test (HOST) was employed to evaluate sperm plasma membrane functionally [47]. In this test, 200 mL of a hypo-osmotic solution—comprising 9 g of fructose and 4.9 g of sodium citrate per liter of distilled water (osmolality: 100 mOsm)—was mixed with 20 μL of the semen sample. After incubating for 60 min at 37 °C, 10 μL of the mixture was placed on a pre-warmed slide and covered with a coverslip. The percentage of sperm with intact plasma membranes was determined by examining 200 sperm across at least five microscopic fields using a phase-contrast microscope at 400× magnification.

Malondialdehyde (MDA) levels were measured using the thiobarbituric acid (TBA) method [48] to assess lipid peroxidation in frozen–thawed sperm cells. First, 1 mL of the sperm sample was mixed with 2 mL of a TBA–trichloroacetic acid (TCA) solution containing 0.375% (*w/v*) TBA, 15% (*w/v*) TCA, and 0.25 N HCl. The mixture was boiled in a water bath at 100 °C for 15 min, then cooled to room temperature. After centrifugation at 1200× *g* for 15 min, the absorbance of the supernatant was measured at 535 nm using a Hitachi U-2001 spectrophotometer (Tokyo, Japan). MDA levels were calculated using the specific absorbance coefficient (1.56 × 10^5^/mol/cm^3^) and expressed in nmol/mL.

The mitochondrial active potential was assessed using the Rhodamine 123 (R123; 83,702) and PI (0.01 mg/mL, 537,060) staining technique [27]. A 300 μL aliquot of diluted semen was treated with 10 μL of Rh123 (0.01 mg/mL) and incubated for 20 min at room temperature in the dark. The samples were then centrifuged at 500× *g* for 3 min and resuspended in 500 μL of Tris buffer. Next, 10 μL of PI (1 μg/mL) was added before flow cytometry analysis. Sperm cells exhibiting a positive signal for Rh123 and a negative signal for PI were recorded as having active mitochondria.

The procedure for assessing sperm hydrogen peroxide (H_2_O_2_) levels [27]. Semen samples were diluted with phosphate-buffered saline (PBS) to a concentration of 3–5 × 10^6^ sperm/mL. Next, 25 μL of the fluorescent dye DCFH-DA (2′,7′-Dichlorfluoresceindiacetate) was added to 1 mL of the sperm suspension, and the mixture was incubated at room temperature for 40 min. After centrifugation to remove the supernatant, the sperm pellet was resuspended in PBS to the same concentration and incubated for an additional 10 min at room temperature. Before flow cytometric analysis, 2 μL of PI (1 μg/mL) was added to the semen samples.

Apoptotic sperm were evaluated using FITC Annexin V (BioLegend, San Diego, CA, USA) to detect phosphatidylserine translocation, with at least 10,000 sperm analyzed per test group. Following the manufacturer’s protocol, the cells were washed twice with cold BioLegend cell staining buffer (Cat. No. 420201); the cells were then suspended in Annexin V Binding Buffer (Cat. No. 422201) at a concentration of 1 × 10^6^ cells/mL. Subsequently, 100 μL of the cell suspension was transferred to a 5 mL test tube, and 5 μL of FITCAnnexin V (Cat. No. 640905) was added to the 100 μL cell suspension and incubated at room temperature for 15 min. Next, 5 μL of PI (Cat. No. 421301) was added, vortexed thoroughly, and further incubated in the dark at room temperature for another 15 min. Finally, 400 μL of Annexin V Binding Buffer was added to each tube, and samples were analyzed by flow cytometry. The sperm subpopulations were categorized as follows: apoptotic cells (Annexin+/PI−), living cells (Annexin−/PI−), and dead cells (Annexin+/PI+).

### 2.4. Statistical Analyses

Data analysis was performed utilizing the Proc GLM procedure in SAS (version 9.1; SAS Institute, Cary, NC, USA) based on a Completely Randomized Design. Orthogonal contrasts were used to compare means, with statistical significance set at *p* < 0.05. The results are presented as mean ± SD.

## 3. Results

The analysis of fresh sperm yielded the following results, presented as mean ± SD: Total motility was observed at 95.35 ± 2.54%, indicating a high level of overall movement. Progressive motility, which refers to the forward movement, was slightly higher at 93.46 ± 2.46%. The percentage of live sperm was 92.32 ± 3.78%. The hypo-osmotic test, assessing sperm membrane integrity, showed results of 90.13 ± 3.24%. Acrosome integrity, crucial for fertilization, was recorded at 89.14 ± 3.75%. Lastly, the proportion of abnormal sperm cells, including those with detached heads, bent tails, looped tails, cytoplasmic droplets, coiled sperm, and neck breakage, was 17.11 ± 7.32%.

The results obtained from CASA revealing the motility and velocity characteristics are detailed in Table 1. The experimental groups demonstrated that the diluents enriched with MitoQ or AFP III alone significantly improved (*p* < 0.05) parameters such as motility rate, progressive motility, VAP, VSL, VCL, ALH, and BCF compared with the extenders combining MitoQ and AFP III. In the control group, diluents containing 0.75 and 1.0 µg/mL AFP III exhibited significantly higher values (*p* > 0.05) than the control, while no significant differences (*p* > 0.05) were observed between the MitoQ group and the control. Notably, motion characteristics, including progressive motility, VAP, VSL, VCL, ALH, and BCF in the 0.75 and 1.0 µg/mL groups, appeared to be superior to those in the AFP and MitoQ combined and control groups. However, no significant differences (*p* > 0.05) were detected in STR, LIN, and WOB among the treated groups when compared to the control.

**Figure 1:**
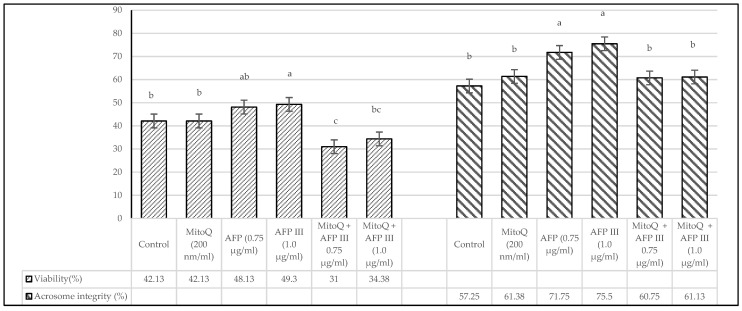
Impact of MitoQ and antifreeze protein III on viability and acrosome integrity of frozen–thawed canine sperm. The SD of the mean values presented in the figure are as follows: viability (4.95, 5.09, 4.66, 5.65, 2.84 and 3.81) and acrosome integrity (3.54, 4.38, 3.54, 2.75, 3.32 and 3.09), respectively, for each treatment from the control to the last (*n* = 8). ^a–c^ Values with different superscripts are different (*p* < 0.05).

**Figure 2:**
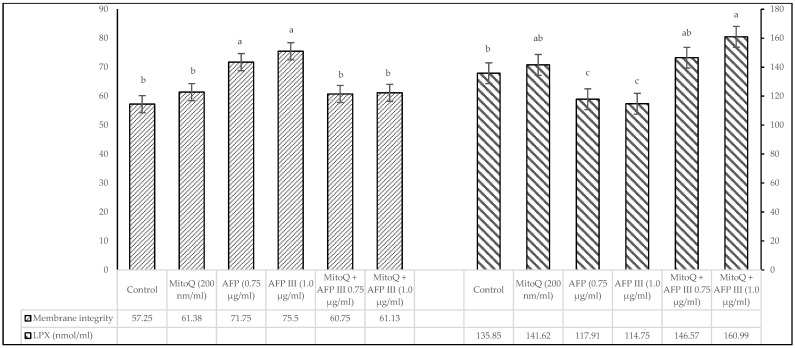
Impact of MitoQ and antifreeze protein III on membrane integrity and lipid peroxidation (LPX) of frozen–thawed canine sperm. The SD of the mean values presented in the figure are as follows: membrane integrity (2.14, 2.71, 2.81, 2.35, 3.39 and 3.00) and LPX (13.53, 12.20, 10.66, 9.60, 9.21 and 8.51), respectively, for each treatment from the control to the last (*n* = 8). ^a–c^ Values with different superscripts are different (*p* < 0.05).

The effects of varying concentrations of MitoQ and AFP III on the viability and acrosome integrity of post-thaw canine sperm are illustrated in Figure 1. The results indicate that diluents supplemented exclusively with AFP III exhibited significant differences (*p* < 0.05) in both viability and acrosome integrity compared with the control and other treatment groups. In contrast, no significant differences were observed between the control and the groups treated with MitoQ and AFP III, except for the diluent containing 1.0 µg/mL of AFP III. The findings related to membrane integrity and lipid peroxidation in cryopreserved canine sperm are presented in Figure 2. The analysis showed statistically significant differences (*p* < 0.05) between the diluents containing 0.75 and 1.0 µg/mL AFP III compared with the other treatment groups. However, no significant differences (*p* > 0.05) were detected between the control group and the treatment groups that incorporated MitoQ alongside varying concentrations of AFP III.

Regarding lipid peroxidation, Figure 2 presents result consistent with those for membrane integrity in frozen–thawed sperm, showing significant differences (*p* < 0.05) in diluents supplemented with 0.75 and 1.0 µg/mL AFP III compared with other treatments and the control group. Furthermore, the extender containing 1.0 µg/mL AFP III tended to have higher values than the treatment with 0.75 µg/mL AFP III.

**Figure 3:**
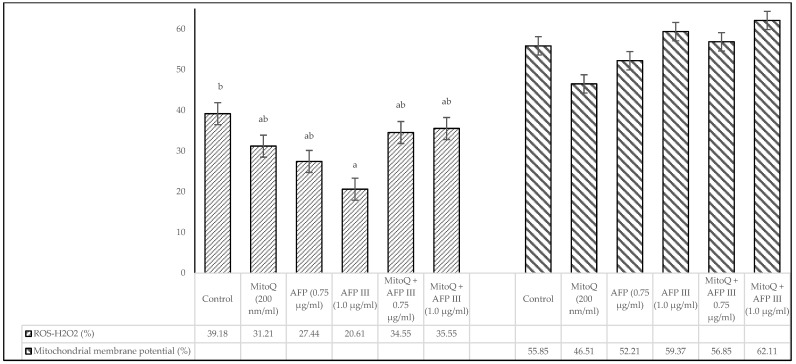
Impact of MitoQ and AFP III on ROS-H_2_O_2_ levels and mitochondrial membrane potential in frozen–thawed canine sperm (*n* = 8). The SD of the mean values presented in the figure are as follows: ROS-H_2_O_2_ levels (2.11, 5.33, 4.02, 2.95, 5.30, 5.29) and mitochondrial membrane potential (8.47, 7.82, 6.85, 7.84, 10.48, 11.10), respectively, for each treatment from the control to the last (*n* = 8). ^a,b^ Values with different superscripts are different (*p* < 0.05).

Figure 3 illustrates the outcomes for intracellular hydrogen peroxide (ROS-H_2_O_2_) levels and mitochondrial membrane potential following the freezing–thawing process. The analysis indicates no statistically significant differences (*p* > 0.05) among the extenders containing MitoQ and AFP III, whether used individually or in combination, when compared to the control group, except for the diluents containing 1.0 µg/mL AFP III, which exhibited significant difference (*p* < 0.05) from the control. However, the experimental groups with 1.0 µg/mL AFP III, either alone or combined with MitoQ, showed a tendency for higher values compared with other treatments and the control.

**Figure 4:**
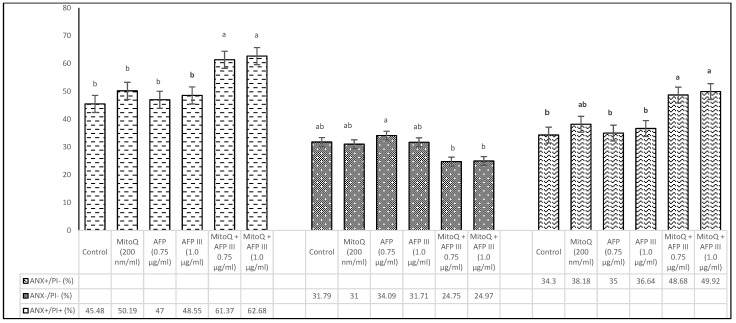
Impact of MitoQ and AFP III on Annexin+/PI− (apoptotic), Annexin−/PI− (living), and Annexin+/PI+ (dead) cells of frozen–thawed canine sperm. The SD of the mean values presented in the figure are as follows: apoptotic cells (3.55, 4.12, 4.30, 5.16, 4.13, 3.78), living cells (3.07, 3.09, 3.24, 3.74, 3.00, 1.85), and dead cells (2.83, 2.86, 2.77, 3.15, 3.67, 3.30), respectively, for each treatment from the control to the last (*n* = 8). ^a,b^ Values with different superscripts are different (*p* < 0.05).

Figure 4 illustrates the results of the phosphatidylserine translocation assay, classifying sperm into three categories: dead (Anx+/Pi+), apoptotic (Anx+/Pi−), and viable (Anx−/Pi−). The analysis was performed using diluents containing MitoQ and AFP III at concentrations of 200 nm/mL and 0.75 or 1.0 µg/mL, respectively. The findings indicate no statistically significant differences (*p* > 0.05) in the proportion of dead sperm between the control group and those treated with MitoQ or AFP III individually. However, a significant difference (*p* < 0.05) was observed when comparing groups treated with combinations of various concentrations of MitoQ and AFP III. Additionally, the data in Figure 4 show a similar effect of MitoQ and AFP III on both viable (Anx−/Pi−) and apoptotic sperm cells. Notably, the diluent containing 0.75 µg/mL AFP demonstrated a significant difference (*p* < 0.05) and exhibited a trend of divergence compared with the other treatments and the control group.

## 4. Discussion

Sperm cryopreservation is associated with numerous stressors, including ice formation, exposure to toxic chemicals, and oxidative stress. The primary challenges include ice injury and cell damage occurring during the phases of ice formation, nucleation, and recrystallization [49,50,51]. In this context, the principal detrimental effects of cryopreservation on sperm quality are fundamentally linked to plasma membrane impairment, leading to mitochondrial dysfunction [52]. Mitochondrial dysfunction caused by cryopreservation negatively affects sperm health by triggering excessive calcium ions release, reactive oxygen species (ROS) production, reduced ATP levels, and cytochrome C release. The incorporation of mitochondrial activators has been shown to enhance the effectiveness of sperm cryopreservation [53]. Consequently, there is an increasing demand in the biomedical sciences for biocompatible cryoprotectants to protect complex biomolecules vital for fertility and reproductive health applications [54,55,56]. MitoQ has emerged as a highly effective mitochondrial antioxidant in cryopreservation media for various species, including bulls, roosters, rams, goats, stallions, and humans [27,28,51,57,58,59,60,61]. On the other hand, antifreeze proteins (AFPs) have evolved in diverse organisms such as bacteria, fungi, crustaceans, microalgae, insects, and fish to adapt to cold environments. These proteins, classified into types I, II, and III, play a crucial role in survival by acting as cryoprotectants and interacting with biological membranes [31,34,35,36,62,63,64]. They maintain cellular membrane integrity, prevent leakage, enhance thermal hysteresis, and inhibit ice formation, thus preserving sperm cells by stabilizing osmotic pressure [43,44]. To the best of our knowledge, this study represents the first investigation into the combined effects of MitoQ and antifreeze protein on the cryopreservation of canine semen. These findings may provide valuable insights to improve the freezing process for sperm cells from various dog breeds.

In this context, reactive oxygen species (ROS) play a crucial role in processes such as chromatin remodeling, hyperactivation, the acrosome reaction, and the fusion of sperm with oocytes. Hydrogen peroxide (H_2_O_2_) is recognized as a significant form of mitochondrial ROS. Elevated H_2_O_2_ levels during sperm cryopreservation have been associated with increased lipid peroxidation (LPO) and subsequent structural damage to sperm [65]. Our findings demonstrate that intracellular H_2_O_2_ levels were significantly reduced in semen samples supplemented with antifreeze protein at 0.75 and 1.0 µg/mL. Furthermore, MitoQ proved effective in lowering H_2_O_2_ concentrations in frozen–thawed sperm, likely due to its ability to enhance mitochondrial antioxidant enzyme activity, particularly superoxide dismutase (SOD). It is well established that SOD plays a vital role in mitigating oxidative stress by neutralizing reactive oxygen species (ROS) and alleviating lipid peroxidation (LPO) and oxidative stress [66,67,68]. Supporting this, previous studies have shown that incorporating MitoQ into ram-sperm-freezing extenders increases SOD activity while reducing ROS and malondialdehyde levels (MDA) [69]. Similarly, in our study, MitoQ likely contributed to reduced mitochondrial ROS by enhancing antioxidant capacity and elevating SOD levels, which in turn resulted in diminished MDA levels as a consequence of LPO in canine frozen–thawed sperm.

The incorporation of MitoQ into the semen medium facilitates its penetration through the inner mitochondrial membrane of sperm cells due to the lipophilic nature of triphenylphosphonium (TPP) cations, resulting in its accumulation within the mitochondrial matrix [68]. Once inside the mitochondria, ubiquinone is absorbed into the mitochondrial membranes following the release of TPP+ [70]. Ubiquinone plays a crucial role in the respiratory chain by accepting two electrons from complex I or II, reducing to ubiquinol, which subsequently transfers electrons to complex III. This process enhances sperm motility by facilitating electron transfer within the electron transport chain and promoting ATP synthesis in the mitochondria [68]. In this study, no significant differences in lipid peroxidation (LPO) were observed between the control group and the diluent supplemented with 200 nm/mL MitoQ, indicating that 200 nm/mL MitoQ is not an optimal concentration for dog semen cryopreservation. However, notable differences were observed between the diluent containing 200 nm/mL MitoQ alone or in combination with antifreeze protein III and other experimental groups. Regarding the membrane integrity, our findings indicate that diluents with antifreeze protein exhibited significantly higher values compared with the control and extenders containing MitoQ alone or in combination with antifreeze protein. Nonetheless, extenders supplemented with MitoQ showed a tendency toward higher membrane integrity values than the control. Furthermore, extenders containing MitoQ effectively mitigated acrosomal damage. The sperm acrosome, which comprises a complex arrangement of membranes and proteins, is particularly susceptible to damage from reactive oxygen species (ROS) [68]. MitoQ has been shown to preserve acrosomal integrity by preventing the oxidative modification of proteins in human spermatozoa [71], consistent with the findings of the present study. These protective mechanisms are essential for minimizing potential mechanical injury to the sperm membrane [72].

Research on mouse spermatozoa has shown that freezing and thawing processes do not significantly affect sperm viability when the concentrations of antifreeze proteins (AFP) in the extender are below 0.1 μg/mL, suggesting limited cryoprotective efficacy at these levels. Consistent with this finding, the AFP concentrations used in the current study are comparable to those reported in previous investigations. However, further comprehensive studies are needed to determine the optimal dosage, potential synergistic effects with other components, various forms of AFP, and the influence of elevated AFP concentrations on cryopreservation efficacy. Our findings demonstrate that groups treated with 0.75 and 1.0 µg/mL AFP in the freezing diluents exhibited a significantly greater number of intact acrosomes. This conclusion is in agreement with other studies [34], which reported an increased proportion of acrosome-intact cells in chimpanzee sperm following cryopreservation with AFP III. This research emphasized the role of AFPs in stabilizing sperm membranes and effectively preventing capacitation and the subsequent acrosome reaction. The acrosomal reaction, a calcium-dependent exocytotic process, is essential for sperm penetration through oocyte membranes [73]. AFPs, also known as thermal hysteresis proteins or ice structuring or binding proteins, function as impermeable cryoprotective agents. Their mechanism involves binding to ice surfaces, which reduces or inhibits the growth of ice crystals [63]. This binding process is critical for preventing ice recrystallization by lowering the freezing temperature below the melting point, a phenomenon known as thermal hysteresis. Additionally, the interaction of antifreeze proteins (AFPs) leads to melting hysteresis, which elevates the melting temperature above its typical value. This mechanism, identified as thermal hysteresis, is critical for the survival of organisms inhabiting extremely cold environments [74].

Extensive research has been conducted on the interactions between AFPs and water or ice crystals at low temperatures, demonstrating their ability to effectively inhibit ice formation and growth through various experimental methodologies [75,76]. The results of this investigation suggest that incorporating AFP III into diluents could significantly advance canine sperm preservation techniques. Moreover, our results indicate that while AFP enhances sperm resilience during freezing, its inclusion in fresh semen does not produce adverse effects. The concentrations used in this study were determined based on prior research, which showed that lower concentrations, such as 0.75 and 1.0 µg/mL, yield optimal outcomes. In contrast, a concentration of 2.0 µg/mL resulted in cytotoxicity when added to freezing diluent, consistent with the existing literature [77,78]. Although further research is needed, the preliminary findings suggest that AFP III concentrations of 0.75 and 1.0 μg/mL may be optimal for canine sperm preservation. At these concentrations, the proportion of slow-moving sperm was significantly different from both the control group and the 2.0 μg/mL concentration. Additionally, this concentration demonstrated improved plasma membrane integrity in relation to AFP III. Enhanced membrane integrity was also noted in canines at concentrations of 0.75 and 1.0 μg/mL of AFP III when compared with the control and the diluent supplemented with 2.0 µg/mL AFP III.

## 5. Conclusions

The incorporation of MitoQ and antifreeze protein III into the extender significantly improves the quality of canine semen. Thus, it can be concluded that MitoQ and antifreeze protein III play a crucial role in minimizing sperm damage, which is essential for the successful cryopreservation of canine semen, particularly in endangered breeds. However, further research is needed to comprehensively evaluate the specific effects of antifreeze protein III on canine sperm.

## Figures and Tables

**Table 1:** Sperm motion characteristics in frozen–thawed canine semen with different concentrations of MitoQ and antifreeze protein III (AFP III) (Mean ± SD).

				Treatments		
	Control	MitoQ 200 nm/mL	AFP III 0.75 µg/mL	AFP III 1.0 µg/mL	MitoQ (200 nm/mL) + AFP III (0.75 µg/mL)	MitoQ (200 nm/mL) + AFP III (1.0 µg/mL)
TM (%)	31.07 ± 4.85 ^ab^	31.34 ± 3.45 ^ab^	37.82 ± 4.76 ^a^	36.99 ± 4.13 ^a^	21.11 ± 2.27 ^c^	25.14 ± 3.84 ^b^
PM (%)	23.14 ± 4.96 ^b^	28.09 ± 4.08 ^ab^	32.53 ± 3.92 ^a^	34.29 ± 4.01 ^a^	20.06 ± 1.54 ^b^	24.30 ± 3.38 ^b^
VAP (µm/s)	21.52 ± 3.53 ^a^	18.32 ± 2.31 ^ab^	21.82 ± 3.04 ^a^	23.28 ± 3.43 ^a^	12.52 ± 1.06 ^b^	12.38 ± 1.98 ^b^
VSL (µm/s)	18.56 ± 3.16 ^a^	15.34 ± 1.78 ^ab^	18.18 ± 2.54 ^a^	19.98 ± 3.01 ^a^	10.70 ± 0.93 ^b^	10.30 ± 1.82 ^b^
VCL (µm/s)	39.24 ± 7.04 ^a^	32.99 ± 4.92 ^ab^	41.65 ± 7.49 ^a^	44.46 ± 7.53 ^a^	22.31 ± 1.96 ^b^	22.74 ± 3.10 ^b^
ALH (µm)	0.44 ± 0.07 ^ab^	0.39 ± 0.05 ^abc^	0.48 ± 0.08 ^a^	0.51 ± 0.08 ^a^	0.30 ± 0.02 ^bc^	0.27 ± 0.04 ^c^
STR (%)	0.86 ± 0.02	0.78 ± 0.07	0.84 ± 0.03	0.86 ± 0.02	0.85 ± 0.01	0.81 ± 0.04
LIN (%)	0.48 ± 0.02	0.48 ± 0.03	0.47 ± 0.04	0.47 ± 0.02	0.48 ± 0.03	0.44 ± 0.05
BCF (Hz)	7.48 ± 0.75 ^ab^	7.33 ± 0.62 ^ab^	7.91 ± 0.66 ^a^	7.97 ± 0.55 ^a^	5.91 ± 0.38 ^b^	5.73 ± 0.79 ^b^
WOB	0.56 ± 0.02	0.57 ± 0.02	0.55 ± 0.03	0.48 ± 0.06	0.57 ± 0.03	0.53 ± 0.04

TM: total motility; PM: progressive motility; VAP: average path velocity; VSL: straight linear velocity; VCL: curvilinear velocity; STR: sperm track straightness; LIN: linearity; ALH: amplitude of lateral head displacement; BCF: beat cross frequency (*n* = 8). ^a–c^ Values with different superscripts in the same row are different (*p* < 0.05).

## Data Availability

The authors retain ownership of the data produced in this study, which can be obtained by contacting the corresponding author.
